# Characterization of workers covered by a risk insurance company in Colombia who suffered amputation

**DOI:** 10.5327/Z1679443520190402

**Published:** 2019-12-01

**Authors:** María Osley Garzón, María Garcés, Daniela Isaza, Susana Jaramillo, Valentina Latorre, Sara Valderrama

**Affiliations:** 1 Doctor in Epidemiology and Biostatistics. Research Teacher. School of Medicine, CES University - Medellín, Colombia. Universidad CES School of Medicine CES University Colombia

**Keywords:** occupational risks, amputation, traumatic, upper extremity

## Abstract

**Background:**

Scarcity of information on the sociodemographic, occupational and clinical characteristics of workers who suffer upper limb amputation hinders planning and implementing actions to improve their living and working conditions.

**Objective:**

To investigate the sociodemographic, occupational and clinical characteristics of workers covered by a risk insurance company in Colombia who suffered upper limb amputations and investigate their association with risk categories.

**Methods:**

Exploratory, descriptive, retrospective and analytical study of data relative to workers covered by a risk insurance company in Colombia who suffered upper limb amputation in the period from 1982 and 2017. Following approval by School of Medicine, CES University, and a risk insurance company, we developed a checklist to collect data on the variables of interest. We performed descriptive and bivariate analysis with 95% of confidence and error of 5%.

**Results:**

The largest proportion of workers who suffered amputation were men aged 36 to 55 years old and residing in the Andean region of Colombia. Amputations mainly followed incidents during the performance of tasks, particularly in jobs in the secondary economic sector and classified as with type 3 risk.

**Conclusion:**

We were able to identify some characteristics associated with accidents, including age, risk class, economic sector, care received and disability duration. The results point to the need for appropriate interventions for the benefit of workers and risk insurance companies.

## RESUMEN |

**Introducción:**

La escasa información sobre las características sociodemográficas, laborales y clínicas de trabajadores que sufren amputaciones de miembro superior dificultan planear y adelantar acciones que aporten al mejoramiento de sus condiciones de vida y de salud.

**Objetivos:**

Determinar las características sociodemográficas, laborales y clínicas de trabajadores con amputaciones en miembros superiores atendidos en una aseguradora de riesgos laborales colombiana, para establecer una asociación con el tipo de riesgo.

**Métodos:**

Estudio exploratorio, descriptivo y retrospectivo con intención analítica. Se tomaron registros de la base de datos de pacientes con amputaciones de miembro superior atendidos en una aseguradora colombiana entre los años 1982 y 2017. Se aplicó una lista de chequeo, previa aprobación del proyecto por parte de la Facultad de Medicina de la Universidad CES y una aseguradora de riesgos laborales colombiana, para identificar las variables en estudio. Se realizaron análisis descriptivos y bivariados utilizando pruebas estadísticas con una confianza del 95% y un error del 5%.

**Resultados:**

Se observó que una mayor proporción de los trabajadores amputados fueron hombres entre 36 y 55 años de edad, residentes y trabajadores en la región andina. Las amputaciones fueron derivadas fundamentalmente de accidentes propios de su labor, particularmente para aquellos del sector secundario, clasificados como riesgo tipo 3.

**Conclusión:**

Se observaron algunas características asociadas a la accidentalidad laboral como edad, clasificación de riesgo, sector económico, tipo de atención hospitalaria requerida y días de incapacidad, evidenciando la importancia de intervenir adecuadamente para el beneficio de los pacientes y la aseguradora de riesgos laborales.

## INTRODUCTION

Work accidents pose a serious problem^1^ inasmuch as they are likely to lead to temporary or permanent disability and might result in amputations - especially of the upper limb - which frequency is estimated to increase in the near future in countries such as the United States^1^^,^^2^^,^^3^^,^^4^. Indeed, following death, accidents are one of the most serious work-related incidents due to their potential to cause incapacity not only to work, but also for activities of daily living. As a result, work accidents represent a true socioeconomic and psychological disaster for the affected workers and their families^1^^,^^3^^,^^4^^,^^5^^,^^6^^,^^7^.

According to the Colombian Ministry of Health and Social Protection, organizations routinely have to deal with workers’ disabilities, which have significant impacts on both employers and health care institutions. Amputation of limbs needed to accomplish tasks further increases costs to organizations, since the overall goal of treatment and rehabilitation is to achieve adequate work reintegration^8^.

Traumatic amputations are the second leading type of amputations among the overall population. However, there are some issues relative to the availability of data, since most concern amputations of the lower limbs. For this reason, gathering scientific evidence relative to risk factors, severity, working conditions, characteristics of accidents and clinical aspects of workers who suffer upper limb amputations is necessary^1^^,^^3^^,^^4^^,^^5^^,^^8^^,^^9^. One of the main problems in this regard in Colombia derives from lack of information on the profile of this population of workers. Research has mostly focused on descriptions of treatments, while the data needed to achieve a better sociodemographic, occupational and clinical characterization have been rather neglected, more particularly in the case of workers covered by risk insurance companies (RIC)^5^^,^^8^^,^^10^.

As a function of the aforementioned considerations, gathering evidence likely to contribute to the design and implementation of prevention programs against work accidents and to improve rehabilitation, as well as to identify some of the - still scarcely investigated - characteristics of traumatic amputations is relevant^11^.

## METHODS

In the present exploratory, descriptive, retrospective and analytical study we analyzed secondary data retrieved from records relative to workers covered by a Colombian RIC who suffered amputation in the period from 1982 to 2017. As per the Colombian Ministry of Health Resolution no. 008430 the present can be categorized as risk-free research and was approved by the research ethics committee of CES University on 6 June 2018, as well as by the involved RIC.

We considered records of workers covered by this RIC, aged above 18 and who suffered amputation. We excluded records with more than 20% of missing data, not available in electronic format or which did not indicate the involved limb.

We designed a checklist including:

- *Sociodemographic variables:* age at the time of the incident, sex and area of residence (one of the nine Colombian political-administrative regions);
- *Occupational variables*: accident setting (workplace or commuting); risk class, categorized as low (activity-related risk types 1 and 2 as per the Colombian Ministry of Labor and Social Security Decree no. 1607/2002)^12^, medium (type 3) or high (types 4 and 5); geographic region; circumstances (entrapment, blows, traffic accidents, falls, exposure to electricity, sharps, etc., as per the just mentioned decree)^13^; and economic sector (primary, secondary, tertiary or temporary jobs)^12^^,^^13^.
- *Clinical and health care variables*: diagnostic category (upper limb or multiple traumatic amputation), main diagnosis (traumatic amputation of: shoulder or arm, elbow or forearm, wrist or hand and/or finger(s), upper limb level unspecified), care setting and work disability duration (in days).

To control for bias we only considered variables with less than 20.0% of missing data and reviewed the checklist format, coherence and accuracy. We performed a previous pilot test with the checklist to assess application, time required for review and data collection, order, clarity and coherence of variables.

### DATA ANALYSIS

We performed descriptive univariate analysis of the distribution of absolute and relative frequencies for qualitative variables. Quantitative variables were subjected to descriptive statistics (measures of central tendency, position and dispersion) and the normal distribution of data was assessed with the K-S test. On bivariate analysis, type of amputation (upper limb: yes/no) was defined as the dependent variable and all other aspects as independent variables. The χ^2^ test was used to investigate association between two dichotomous or polytomous qualitative variables. Strength of association was expressed as prevalence ratio (PR) and corresponding 95% confidence interval (95%CI). The relationship between the dependent and quantitative independent variables, as e.g. age and work disability duration, was assessed by means of the Mann-Whitney U test. All the tests were performed with 95% confidence level and error of 5%. The data were processed using software Epidat version 3.1, Excel 2013 and Word 2013.

## RESULTS

**Figure 1.**
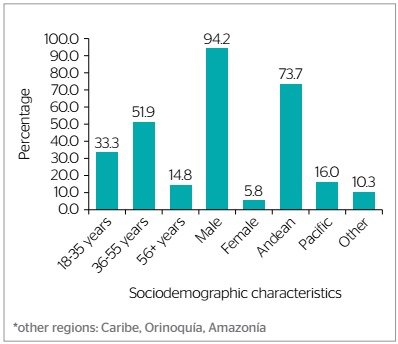
Demographic characteristics of workers covered by a risk insurance company who suffered amputations in the period from 1982 to 2017, Colombia (n=243)

A total of 245 workers covered by the involved RIC suffered amputation in the period from 1982 to 2017. Most of these workers were male (94.0%; n=229) as shown in Figure 1. The highest prevalence of amputations corresponded to workers aged 36 to 55 years old (51.8%; n=126) followed by those aged 18 to 35 (33.5%; n=81) (Figure 1). Most of the affected workers (74.0%) resided in the Andean region of Colombia (Figure 1).

About 90.0% of the incidents leading to amputation occurred during the performance of job tasks. Most incidents involved entrapment, blows or being stepped on (80.4%, n=197) followed by exposure to/contact with electricity. Most cases corresponded to tasks classified as with risk type 3, i.e. medium risk (52.2%) and about one fourth (24.5%) as high risk (types 4 and 5). Almost half of the workers who suffered amputation (46.9%) worked in the secondary economic sector and 6.9% in the primary sector (Table 1).

**Table 1.** Occupational and accident characteristics relative to workers covered by a Colombian risk insurance company who suffered amputations in the period from 1982 to 2017, Colombia (n=243)

Variable	n	(%)
Incident setting		
Workplace	215	(88.5)
Commuting	28	(11.5)
Type of incidente		
Entrapment/blows/being stepped on	197	(80.4)
Falls from Heights > 1.5 m	3	(1.2)
Exposure to/contact with electricity	16	(6.5)
Contact with tools/sharps	12	(4.9)
Otro	17	(6.9)
Risk class		
4-5	60	(24.5)
3	128	(52.2)
1-2	57	(23.3)
Geographic region		
Andean	174	(71)
Pacific	42	(17.1)
Other*	29	(11.8)
Economic sector		
Primary	17	(6.9)
Seondary	115	(46.9)
Tertiary	70	(28.6)
Temporary job	43	(17.6)

*Caribe, Orinoquía, Amazonía

### CLINICAL ASPECTS OF WORKERS WHO SUFFERED AMPUTATION

About 82.4% of the workers (n=202) suffered traumatic amputation of the upper limb (shoulder, arm, forearm, wrist and/or hand). Amputations involving the wrist or hand/finger(s) were the most frequent (48.2%) followed by those at the level of the elbow or forearm (20.4%). Most workers received in-hospital surgical treatment (69.8%) or in an intensive care unit (20.4%). The largest proportion of workers remained disabled 185 to 365 days (42.9%) (Table 2).

**Table 2.** Clinical characteristics of workers covered by a Colombian risk insurance company who suffered amputations in the period from 1982 to 2017, Colombia (n=243)

Variables	n (%)
Diagnostic category	
Upper limb traumatic amputation	202 (82.4)
Traumatic amputation of other body parts	43 (17.6)
Main diagnosis (traumatic amputation)	
Shoulder or arm	31 (12.7)
Elbow or forearm	50 (20.4)
Wrist or hand/finger(s)	118 (48.2)
Upper limb, unspecified level	3 (1.2)
Other limb	43 (17.6)
Main health care approach	
Intensive care	50 (20.4)
In-hospital surgery	171 (69.8)
Outpatient surgery	14 (5.7)
In-hospital	5 (2)
Outpatient	5 (2)
Disability duration (days)	
>365	60 (24.5)
181-365	105 (42.9)
91-180	63 (25.7)
31-90	15 (6.1)
None	2 (0.8)

### SOCIODEMOGRAPHIC CHARACTERISTICS ASSOCIATED WITH UPPER LIMB AMPUTATION

**Figure 2.**
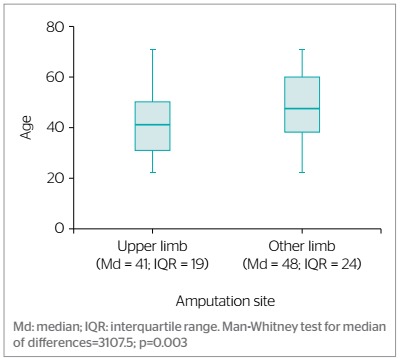
Site of amputations recorded by a risk insurance company according to workers’ age, Colombia, 1982-2017 (n=245)

As shown in Figure 2, the workers who suffered upper limb amputation were significantly younger than those who underwent other types of amputation (p=0.003). There was statistically significant relationship between higher prevalence of upper limb amputation and younger age (p=0.000). The prevalence of this type of amputation was 53.0% higher among the workers aged 18 to 35 compared to those aged 56 or older (PR=1.53; 95%CI 1.15; 2.03) and 46.0% higher among those aged 36 to 55 (PR=1.46; 95%CI 1.09; 1.94) (Table 3).

**Table 3.** Association between sociodemographic characteristics and amputation site relative to workers covered by a Colombian risk insurance company, 1982 to 2017, Colombia (n=243)

Variables	Amputation site n (%)		Total	χ^2^ (p)	PR (95%CI)*
	Upper limb	Other			
Age					
18-35	73 (36.1)	9 (20.9)	82 (33.5)	17.43 (0.000)	1.53 (1.15; 2.03)
36-55	108 (53.5)	19 (44.2)	127 (51.8)		1.46 (1.09; 1.94)
56+	21 (10.4)	15 (34.9)	36 (14.7)		1.00
Sex					
Male	191 (94.6)	40 (93)	231 (94.3)	0.001 (0.975)	1.02 (0.93; 1.11)
Female	11 (5.4)	3 (7)	14 (5.7)		1.00
Geographic region					
Andean	154 (76.2)	26 (60.5)	180 (73.5)	10.88 (0.004)	0.97 (0.83; 1.12)
Pacific	25 (12.4)	14 (32.6)	39 (15.9)		0.72 (0.55; 0.95)
Other*	23 (11.4)	3 (7)	26 (10.6)		1.00

PR: prevalence ratio; 95%CI: 95% confidence interval. Value 1 indicates the reference category; *Caribe, Orinoquía, Amazonía

We found statistically significant association (p=0.004) between residing in the Pacific region and lower prevalence of upper limb amputation. The prevalence of upper limb amputation was 28.0% lower (PR=0.72; 95%CI 0.55; 0.95) for the workers who lived in the Pacific region compared to other regions and 25% lower than that of workers who lived in the Andean region. We did not find any difference in prevalence as a function of sex (Table 3).

### OCCUPATIONAL AND ACCIDENT CHARACTERISTICS ASSOCIATED WITH UPPER LIMB AMPUTATION

We found statistically significant association (p<0.05) between upper limb amputation and incident setting, economic sector and geographic region of residence (Table 4). The prevalence of upper limb amputation was 83.0% higher among the workers who were involved in accidents while performing job tasks (PR=1.83; 95%CI 1.37; 2.45) compared to commuting accidents. Prevalence was 39.0 and 31.0%, respectively, higher among the workers engaged in the secondary economic sector (PR=1.39; 95%CI 1.16; 1.66) or had a temporary job (PR=1.31; 95%CI 1.06; 1.61) (Table 4). In turn, prevalence was lower (22.0%) for the workers in the Pacific (PR=0.78; 95%CI 0.62; 2.45) compared to the Andean region (Table 4).

**Table 4.** Association between occupational and accident characteristics and amputation site relative to workers covered by a Colombian risk insurance company, 1982 to 2017, Colombia (n=243)

Variables	Amputation site n (%)		Total	χ^2^ (p)	PR (95%CI)*
	Upper limb	Other			
Incident setting					
Workplace	193 (96)	22 (52.4)	215 (88.5)	64.89 (0.000)	1.83 (1.37; 2.45)
Commuting	8 (4)	20 (47.6)	28 (11.5)		1.0
Risk class					
4-5	47 (23.3)	13 (30.2)	60 (24.5)	4.87 (0.087)	1.04 (0.85; 1.26)
3	112 (55.4)	16 (37.2)	128 (52.2)		1.16 (0.98; 1.4)
1-2	43 (21.3)	14 (32.6)	57 (23.3)		1.0
Economic sector					
Primary	14 (6.9)	3 (7)	17 (6.9)	14.46 (0.000)	1.25 (0.95; 1.65)
Secondary	105 (52)	10 (23.3)	115 (46.9)		1.39 (1.16; 1.66)
Temporary job	37 (18.3)	6 (14)	43 (17.6)		1.31 (1.06; 1.61)
Tertiary	46 (22.8)	24 (55.8)	70 (28.6)		1
Geographic region					
Andean	149 (73.8)	25 (58.1)	174 (71)	8.69 (0.012)	1.0
Pacific	28 (13.9)	14 (32.6)	42 (17.1)		0.78 (0.62; 0.97)
Other*	25 (12.4)	4 (9.3)	29 (11.8)		1.00 (0.86; 1.18)

PR: prevalence ratio; 95%CI: 95% confidence interval. Value 1 indicates the reference category; *Caribe, Orinoquía, Amazonía

Although statistically non-significant, the prevalence of upper limb amputation was 16.0% higher among the workers who performed type 3 risk tasks (Table 4).

We found statistically significant difference (p<0.05) for variables healthcare modality and disability duration. The prevalence of outpatient (PR=1.67; 95%CI 1.33; 2.09) and inpatient (PR=1.48; 95%CI 1.17; 1.87) surgical intervention was 67.0 and 48.0% higher than that of intensive care, respectively. This difference was statistically significant (p=0.000) (Table 5). Although statistically non-significant, the prevalence of outpatient care was 33.0% higher compared to intensive care (Table 5).

**Table 5.** Association of health care modality and disability duration with amputation site relative to workers covered by a Colombian risk insurance company, 1982 to 2017, Colombia (n=243)

Variables	Amputation site n (%)		Total	χ^2^ (p)	PR (95%CI)*
	Upper limb	Other			
Health care					
Intensive care	30 (14.9)	20 (46.5)	50 (20.4)	31.41 (0.000)	1.00
In-hospital surgery	152 (75.2)	19 (44.2)	171 (69.8)		1.48 (1.17; 1.87)
Outpatient surgery	14 (6.9)	-	14 (5.7)		1.67 (1.33; 2.09)
In-hospital	4 (2)	1 (2.3)	5 (2)		1.33 (0.81; 2.18)
Outpatient	2 (1)	3 (7)	5 (2)		0.67 (0.22; 1.99)
Disability duration (days)					
>365	38 (18.8)	22 (51.2)	60 (24.5)	23.97 (0.000)	1.00
181-365	90 (44.6)	15 (34.9)	105 (42.9)		1.35 (1.09; 1.66)
91-180	59 (29.2)	4 (9.3)	63 (25.7)		1.48 (1.20; 1.81)
31-90	14 (6.9)	1 (2.3)	15 (6.1)		1.47(1.16; 1.86)
None	1 (0.5)	1 (2.3)	2 (0.8)		0.79(0.19; 3.20)

PR: prevalence ratio; 95%CI: 95% confidence interval. Value 1 indicates the reference category.

**Figure 3.**
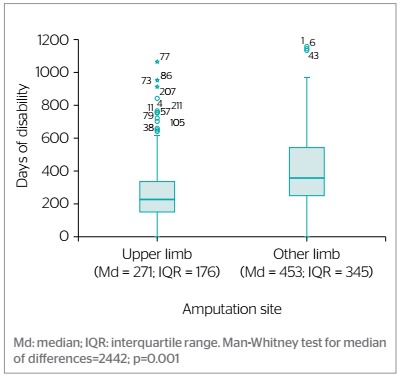
Disability duration for workers covered by a risk insurance company who suffered amputations in the period from 1982 to 2017 according to amputation site, Colombia, 1982-2017 (n=243)

Disability was shorter for the workers who suffered upper limb compared to lower limb amputation, 271 (interquartile range: 171, p<0.001) versus 453 days, as shown in Figure 3. Overall, disability lasted less than 365 days for the workers who suffered upper limb amputation. In the comparison with disability lasting more than 365 days, differences in prevalence were as follows: 92-180 days, 48.0% higher (PR=1.48; 95%CI 1.20; 1.81); 31-90 days, 47% higher (PR=1.47; 95%CI 1.16; 1.86); and 181-365 days, 35.0% higher (PR=1.35; 95%CI 1.09; 1.66) (Table 5).

## DISCUSSION

### SOCIODEMOGRAPHIC CHARACTERISTICS

The frequency of amputations was higher among younger workers (84.2% 18 to 55 years old) with more than 50% corresponding to workers aged 36 to 55. These findings agree with those reported in a study performed in Illinois, USA, in which 74.0% of amputations corresponded to workers aged 24 to 54. Barouti^14^ found that two-thirds of amputations corresponded to workers under 40, as was also reported by Maryland and other authors^4^^,^^14^^,^^15^^,^^16^.

As in other studies^1^^,^^4^^,^^7^^,^^14^^,^^15^^,^^16^^,^^17^ also in the present one amputations were most frequent among men, 94.0%, thus a rate similar to that reported by Camacho-Conchucos^1^, 98.2%, but higher than that in the aforementioned study performed in Illinois^15^, 88.8%. These findings suggest that men still perform high-risk jobs liable to lead to amputation. As possible reasons, female workers might not have been yet included in all production activities or are more careful in jobs with higher risk of accidents. Priya Varma et al.^4^ observe that in addition to age, also sex (biological condition) has a considerable role in traumatic amputations in general, since in their study risk of amputation was twice higher for the men. However, we did not find significant association between sex and prevalence of upper limb amputations. Also differing from Priya Varma et al.^4^ in our study the prevalence of amputation was highest (53.0%) for the youngest workers (18 to 35 years old) followed by the group aged 36 to 55 (46.0%).

The smallest proportion of workers who suffered upper limb amputation (28.0%) resided in the Pacific region (p<0.05). We were not able to locate any study that considered the place of residence of workers who underwent amputation.

### OCCUPATIONAL CHARACTERISTICS

Most incidents leading to amputation took place during the performance of job tasks (88.5%) and only a smaller fraction while commuting. This rate is similar to that reported by Whelan et al.^18^, 85.5%. Incidents mostly involved entrapment, blows or being stepped on (80.4%) as also in the study by Camacho-Conchucos^1^ in which the most common accidents involved entrapment (58.3%) followed by blows (12%).

Most incidents leading to upper limb amputation occurred within the secondary economic sector (46.9%) which precisely includes the occupations most exposed to entrapment, blows or being stepped on. In Camacho-Conchucos’ study^1^ the highest rate of amputations corresponded to the manufacturing sector (37.9%) therefore similar to our findings. However, he did not detail the activities included in this sector, therefore we cannot assert whether they were or not exactly the same as the ones we considered. Diverging from both Camacho-Conchucos^1^ and our findings, in a study performed in Asturias, Spain^5^ most workers who suffered amputation worked within the primary economic sector, versus 6.9% in our study.

We were not able to locate any study that analyzed variables geographical region and risk class for the purpose of comparison. The reason might derive from the particularities of the political-administrative division of Colombia and the risk classification used in the country, based on the Decree no. 1607 from 2002^12^. In our study 73.5% of the workers who suffered amputation lived in the Andean region (n=180), most (52.2%) had medium-risk jobs (class 3) and one fourth high-risk jobs (classes 4 and 5) as per the categories established in the just mentioned decree^12^.

We found statistically significant association (p=0.000) between upper limb amputation and economic sector. Prevalence was higher for workers in the secondary (39.0%) and primary (25.0%) sectors or had temporary jobs (31.0%) compared to the tertiary sector.

### CLINICAL CHARACTERISTICS

Evidence in the literature indicates that most amputations involve the lower limbs^4^^,^^19^^,^^20^. However, in several studies more than 50.0% of amputations related to work accidents involved the upper limbs, 30.0% the lower limbs and 12% more than one limb^16^^,^^17^. In the present study, the prevalence of upper limb amputations was 30.0% higher, 82.4% (n=202) of them corresponding to traumatic amputations. This is a highly relevant finding, while this type of amputation might be related to risk categories and how job tasks are actually performed.

A considerable proportion of the workers who were victims of accidents (20.3%) required intensive care and 70.0% in-hospital surgical interventions. We found statistically significant association (p=0.000) between main health care approach and upper limb amputations. The latter’s prevalence was higher among the workers who required outpatient (67.0%) or in-hospital (48.0%) surgical care. The prevalence of upper limb amputations was also associated (p=0.000) with disability duration. Prevalence was higher for the workers who remained disabled 91-180, 31-90 and 19-365 days. However, this type of information is still scarcely reported in the literature and this fact hindered our attempts at comparing results.

In a study performed in Spain^5^ 70% of amputations were transradial and 30% transhumeral. In the study by Tennent et al.^19^ transradial amputations corresponded to 47% of the total, the transhumeral level to 34%, wrist disarticulation to 13%, shoulder disarticulation to 4%, while elbow disarticulation was the least frequent (1%). In the study by Chul Ho Jang et al.^20^ transradial amputations represented 51.9% of the cases, followed by transhumeral amputations (32.8%) and shoulder disarticulation (8.2%). Our findings therefore differ, since amputations at the level of the wrist or hand/finger(s) (48.2%) or of the elbow or forearm (20.4%) were the most common.

Among the limitations of the present study, the source of information hindered analysis relative to possible variables, as well as the availability of data. We did not have access to variables representing all factors potentially associated with the analyzed type of amputations, in addition to the scarce published information on this subject and the period selected for the study, i.e. more than two decades.

## CONCLUSION

Despite its limitations and difficulties in the development of the present study, we stress its significance as an onset of efforts to gather scientific evidence from the available information to thus facilitate later studies. More thorough studies are needed to establish the forms of and circumstances under which work accidents occur, with the known consequences for the lives and happiness of workers and their families, to thus ground actions from the perspective of workplace health promotion rather than centered on secondary and tertiary prevention, as was the case of the workers analyzed in the present study.
